# Efficacy and Safety of Peroral Endoscopic Myotomy for Sigmoid-Type Achalasia: A Systematic Review and Meta-Analysis

**DOI:** 10.3389/fmed.2021.677694

**Published:** 2021-07-08

**Authors:** Jin Xu, Chunyu Zhong, Shu Huang, Xinyi Zeng, Shali Tan, Lei Shi, Yan Peng, Muhan Lü, Lianjun Ma, Xiaowei Tang

**Affiliations:** ^1^Department of Gastroenterology, Affiliated Hospital of Southwest Medical University, Luzhou, China; ^2^Department of Gastroenterology, the People's Hospital of Lianshui, Huaian, China; ^3^Department of Endoscopy Center, China-Japan Union Hospital of Jilin University, Jilin University, Changchun, China

**Keywords:** sigmoid-type achalasia, peroral endoscopic myotomy, systematic review, meta-analysis, achalasia

## Abstract

**Background:** The efficacy and safety of peroral endoscopic myotomy (POEM) in the treatment of sigmoid-type achalasia is unknown. This meta-analysis aims to explore the clinical outcomes of POEM for sigmoid-type achalasia.

**Method:** We searched all relevant studies published up to September 2020 in PubMed, Embase, and Cochrane library databases. Meta-analyses for clinical success, Eckardt score, angle of esophageal tortuosity, diameter of esophagus, lower esophageal sphincter (LES) pressure, integrated relaxation pressure (IRP), adverse events, and gastroesophageal reflux diseases were performed based on random or fixed-effects models as needed.

**Results:** We found a total of eight studies that provided data on 248 patients. Overall, the pooled clinical success was achieved in 211 sigmoid-type achalasia patients [90.4%; 95% confidence interval (CI), 85.5%−93.8%]. The pre- and post-POEM Eckardt scores, angle of esophageal tortuosity, diameter of esophageal, LES pressure, and IRP were significantly improved (All *p* < 0.05). The pooled adverse events rate was 13.0% (95% CI, 3.6%−37.4%). The pooled objective confirmation of reflux rate was 41.5% (95% CI, 26.5%−58.3%), and symptomatic reflux rate was 12.5% (95% CI, 8.3%−18.4%).

**Conclusions:** Our current evidence indicated that POEM is an effective and safe therapeutic modality for the treatment of sigmoid-type achalasia.

## Background

Achalasia is an idiopathic esophageal dyskinetic disorder, which is characterized by aperistalsis of the esophageal body and failure of relaxation of the lower esophageal sphincter (LES) (1). It is a rare disease with an estimated prevalence of 10–15.7 per 100,000 inhabitants and an incidence of 1.07–2.2 cases per 100,000 inhabitants/year (2). Sigmoid-type esophagus is the end-stage of achalasia featured by significant dilation and tortuous of the esophageal body leading to a sigmoid-type appearance (3). Sigmoid achalasia may develop in up to 10% of patients with a history of achalasia more than 10 years (4). With the deterioration of achalasia, patients usually experience progressive dysphagia, frequent aspiration, weight loss, and cachexia (5).

Unfortunately, no treatment can restore normal esophageal function. Accordingly, the aim of treatments is to reduce the LES pressure. However, the treatment of sigmoid-type achalasia is still controversial. Endoscopic interventional therapy, such as pneumatic dilatation (PD) and botulinum toxin injection (BTI), are considered invalid (6). Historically, esophagectomy or laparoscopic myotomy was considered the primary treatment of choice for sigmoid-type patients (7–9). Nevertheless, it was an invasive method with high risk of perioperative morbidity and mortality (7, 8, 10). Currently, peroral endoscopic myotomy (POEM) has become the standard treatment for achalasia worldwide because it was minimally invasive and has a higher efficacy than traditional therapeutic methods (11). However, the dilated, swerved, and rotated tortuous esophageal body may make POEM more technically challenging. Nowadays, some researchers have reported the promising results of POEM in sigmoid-type achalasia (6, 12–18). Therefore, we conducted this systematic review and meta-analysis aiming to explore the clinical outcome of POEM for sigmoid-type achalasia.

## Methods

### Search Strategy

The study was conducted in accordance with the Preferred Reporting Items for Systematic Reviews and Meta-analyses (PRISMA) recommendations (19). A comprehensive literature research up to September 2020 was performed by two independent investigators to identify the English-written studies on POEM for the treatment of sigmoid-type achalasia. PubMed, Embase, and Cochrane databases were searched using the term “achalasia” and “POEM.” Our search did not include the word “sigmoid-type achalasia” to ensure a comprehensive search for literature available to POEM (Supplementary Table 1).

### Inclusion and Exclusion Criteria

We included case series and cohort studies which satisfied our inclusion criteria: (1) population: patients were diagnosed with sigmoid-type achalasia; (2) intervention: POEM; and (3) outcome: technical success, clinical success, Eckardt score, angle of esophageal tortuosity, diameter of esophageal, LES pressure, integrated relaxation pressure (IRP), adverse events rate, and gastroesophageal reflux diseases. The exclusion criteria included the following: (1) studies were not written in English, (2) animal studies; (3) case reports with <3 patients; (4) reviews or commentaries; (5) no data for meta-analysis; and (6) overlapping publications.

### Data Extraction and Definition

Two authors individually extracted data from eligible studies. Disagreements were resolved by discussion between the two review authors. If agreement is still not reached, it was up to the third author to decide. Analyzed data included the following: (1) baseline characteristics of studies: first author, year of publication, country, study duration, study design, number of patients, age, gender, duration of symptom, previous interventions, and sigmoid type; (2) clinical outcomes of studies: myotomy length, procedure time, hospital stay, technical success, clinical success, pre- and post-POEM Eckardt score, angle of esophageal tortuosity, diameter of esophageal, LES pressure, IRP, and follow-up time; and (3) adverse events and gastroesophageal reflux diseases after POEM.

Sigmoid-type achalasia was subdivided into sigmoid type 1 (S1) and sigmoid type 2 (S2) according to the degree of tortuosity of the esophageal lumen seen at barium swallow and/or CT scan. In S1, the esophagus was significantly dilated and tortuous but only a single lumen was seen on CT; in S2, the esophagus was very dilated and severely tortuous with U-turns in a proximal direction and a double lumen was identified on some CT slices (6). The other classification included sigmoid type (Sg) and advanced sigmoid type (aSg). Sg was diagnosed when the long axes of the lower esophagus crossed at an angle of 90°-135°, and the aSg was diagnosed when the angle was below 90° (14). Technical success was defined as completion of the whole POEM procedure. The clinical success was defined as a reduction in Eckardt score to ≤ 3 at the follow-up assessment. Adverse events were defined as events requiring additional intervention during or after POEM procedure. Gas-related events without obvious clinical symptoms and further intervention were not considered adverse events.

### Assessment of Study Quality

The two authors individually assessed the quality of the included studies using the Newcastle-Ottawa-Scale (NOS) quality assessment tool (20). The scale ranges between zero up to nine stars, categorized into three dimensions: selection, outcome, and comparability. Stars ≥5 were regarded as high-quality literature.

### Statistical Analysis

The meta-analysis was carried out using the Comprehensive Meta-Analysis software version 2 and Review Manager. *p* < 0.05 was indicated statistically significant. The incidence of clinical success, adverse events, and gastroesophageal reflux diseases in each study was combined, to yield a pooled rate with a 95% confidence interval (CI) for all studies. For meta-analyses of continuous variables, involving Eckardt score, angle of esophageal tortuosity, diameter of esophageal, LES pressure, and IRP, the effect size was represented as a mean difference (MD) and 95% CI. If the study data was expressed as median and interquartile range (IQR) or range, it was converted to mean and standard deviation (SD) using the Luo et al. (21) and Wan et al. (22) formula before analysis. Statistical heterogeneity was examined using the *I*^2^ statistics. We considered *I*^2^ higher than 50% to represent considerable heterogeneity (23). A random-effects model was applied when heterogeneity was considered. Otherwise, the fixed-effects model was adopted. Sensitivity analysis was conducted to assess the influence of each individual study on pooled results. In addition, the funnel plots were utilized to evaluate publication bias in the study.

## Results

### Study Selection

**Figure 1:**
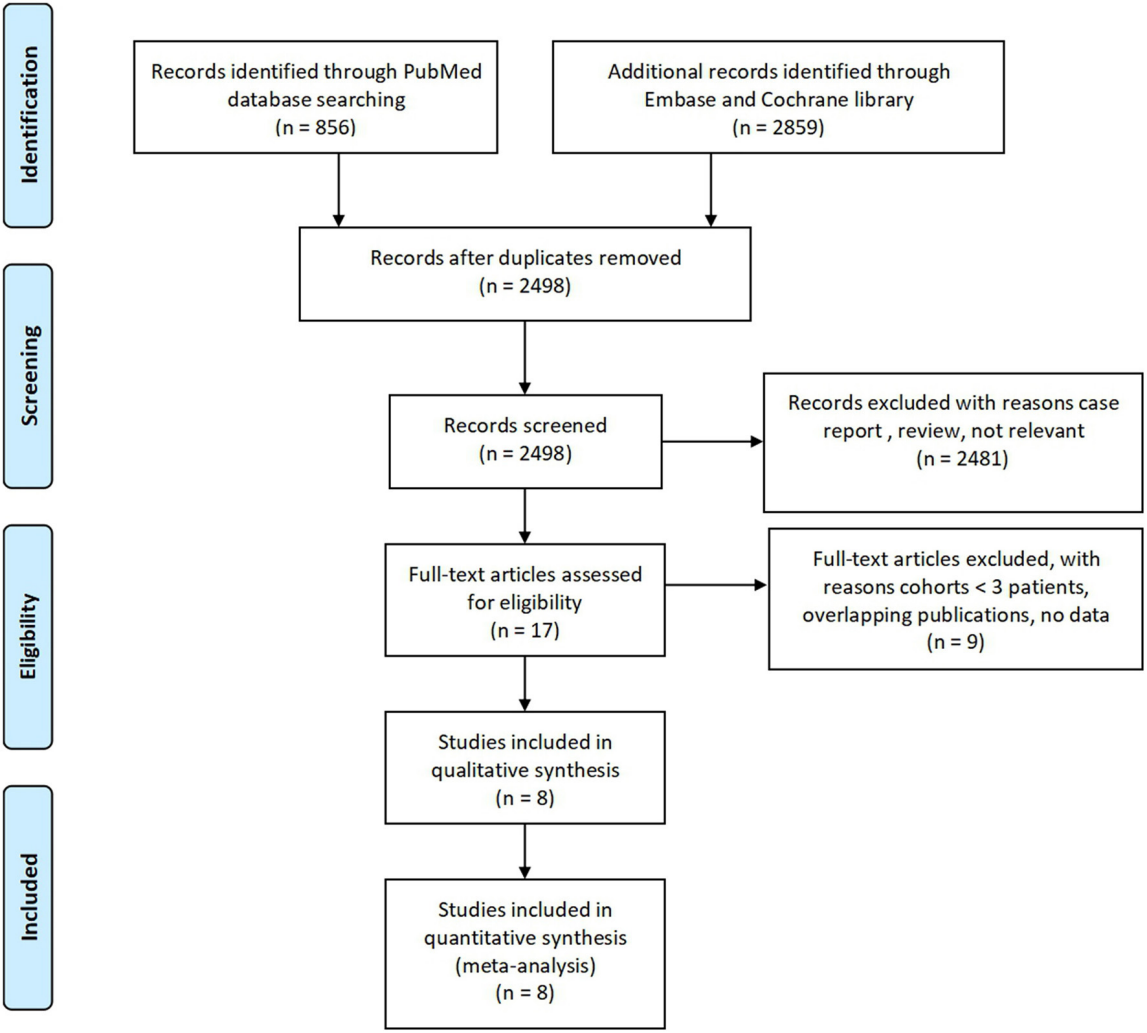
PRISMA flow chart.

A PRISMA flow chart of this systematic review is shown in Figure 1. In summary, a total of 3,715 citations were identified using the described literature search strategy. After the removal of duplicate publications, 2,498 studies were screened for compliance with the eligibility criteria. After reviewing the titles and abstracts, 17 studies were retrieved as full text. Of these, eight studies met the inclusion criteria. Finally, the eight articles were included in our meta-analysis.

### Characteristics of Included Studies

Demographic and case characteristics of patients included in the analysis are provided in Table 1. Overall, all studies were published between 2015 and 2020. Of these, three studies were performed in China, two in Japan, one in Korea, one in USA, and one in India. All the articles had a retrospective design except for one prospective study by Hu et al. (6). The number of patients studied in the included studies ranged from 4 to 108 patients, and the sum of all sigmoid-type achalasia patients was 248. Among them, 44.25% of the enrolled patients were female, and the median of the mean ages from all studies was 51 years (range: 39–63 years). The median of the mean duration of symptoms was 17 months (range: 3–166 months). The rate of previous interventions was 56.9% (*n* = 141). Ninety-seven patients had undergone PD, 15 patients had prior Heller myotomy, 11 patients had undergone BTI, and 18 patients had other interventions.

**Table 1:** The baseline characteristics of included studies.

**Study**	**Year of publication**	**Country**	**Duration**	**Study design**	**Patient (*n*)**	**Age (years)**	**Gender (M/F)**	**Duration of symptoms (months)**	**Previous interventions**	**Sigmoid type**
Hu et al. (6)	2015	China	Nov 2010–Jul 2012	Prospective	32	43.6 (range 18–72)	17/15	3.4 (range 0.1–50)	PD 14; stent 3; BTI: 3; HM 3	S1/S2: 29/3
Tang et al. (12)	2015	China	Jul 2012–Aug 2013	Retrospective^*^	4	39.8 ± 6.8	4/0	11 (range 3–20)	PD 1	–
Lv et al. (13)	2016	China	Aug 2011–Jun 2014	Retrospective	23	49^∧^ (range 21–72)	5/18	96^∧^ (range 24–300)	PD 6; Stent 1; BTI 2; HM 1	S1/S2: 19/4
Maruyama et al. (14)	2020	Japan	May 2015–Dec 2017	Retrospective	16	63.4 ± 15.4	12/4	–	PD 5	Sg/aSg: 11/5
Yoon et al. (15)	2020	Korea	Jul 2013–Dec 2018	Retrospective	13	53.3 (range 17–81)	7/6	165.7 (IQR 228)	PD 5	Sg/aSg: 8/5
Fujiyoshi et al. (16)	2020	Japan	Sept 2008–Jun 2019	Retrospective^*^	108	58.4 ± 14.7	57/51	17.4 (range 7.7–29)	PD 49; Hellor-Dor 8; HM 2	–
Sanaka et al. (17)	2020	United States	Apr 2014–Dec 2019	Retrospective	20	63.3^∧^ (IQR 55.5–72.4)	13/7	5.0^∧^ (IQR 2.0–13.0)	PD 4; BTI 6; HM 6; PD+BTI 1; CRE balloon and savory dilation 5	–
Nabi Z et al. (18)	2020	India	Dec 2014–Nov 2018	Retrospective	32	43.84 ± 13.29	23/9	166.40 ± 44.77	PD 13; HM 3	–

*M/F, male/female; PD, pneumatic dilatation; BTI, botulinum toxin injection; HM, Heller myotomy; Sg/aSg, sigmoid type/advanced sigmoid type; IQR, interquartile range; CRE, controlled radial expansion*.

* *Published conference abstracts*.

∧ *Median*.

### Outcome

The clinical outcomes of included studies are shown in Table 2. Myotomy length of POEM procedure was reported in all but two series, which range from 5.3 to 11.7 cm. The procedure time was available in all but one series. The median of the mean procedure time was 67.6 min (range: 55.3–95.9 min). The hospital stay was also reported in all but two series. The median of the mean hospital stay was 4.5 days (range: 1–6.9 days).

**Table 2:** The clinical outcomes of included studies.

**Study**	**Myotomy length (cm)**	**Procedure time (min)**	**Hospital stay (days)**	**Technical success**	**Clinical success**	**Eckardt score (pre/** **post-POEM)**	**Angle of esophageal tortuosity** ** (pre/** **post-POEM)**	**Diameter of esophageal (mm) (pre/** **post-POEM)**	**LES pressure (mmHg)** ** (pre/** **post-POEM)**	**IRP (mmHg) (pre/** **post-POEM)**	**Follow-up (months)**
Hu et al. (6)	E 8.0 (range 5–11) G 2.3 (range 2–5) T 10.3 (range 7–14)	63.7 (range 22–130)	3.9 (range 1–29)	32/32 (100%)	30/31 (96.8%)	7.8 (range 4–12)/ 1.4 (range 0–5)	–	–	37.9 (range 21.9–70.3)/ 12.9 (range 7.7–22.5)	–	30.0 (range 24–44)
Tang et al. (12)	5.3 (range 5–6)	55.3 (range 45–70)	5.8 ± 2.2	4/4 (100%)	4/4 (100%)	–	–	–	–	–	12
Lv et al. (13)	–	67.6 (range 45–120)	5^∧^ (range 3–10)	23/23 (100%)	22/23 (95.6%)	7^∧^ (range 4–11)/1	–	58.2 ± 11.6/ 37.5 ± 7.3	34.78 ± 4.51/ 11.50 ± 2.56	29.52 ± 3.67/ 10.61 ± 1.54	18^∧^ (range 12–42)
Maruyama et al. (14)	E 8.6 ± 2.5 G 3.1 ± 1.2 T 11.7 ± 2.5	94.7 ± 31.4	6.9 ± 3.4	16/16 (100%)	16/16 (100%)	4.9 ± 2.1/ 0.4 ± 0.6	88.4 ± 23.1/ 109.5 ± 16.7	–	19.4 ± 10.2/ 9.2 ± 6.4	17.6 ± 9.2/ 7.9 ± 5.5	2
Yoon et al. (15)	–	–	–	13/13 (100%)	13/13 (100%)	7.0 (range 4–10)/0.5 (range 0–2)	91.5 ± 13.9/ 114.6 ± 17.5	67.6 ± 27.5/ 49.8 ± 18.0	–	17.5 ± 7.8/ 8.8 ± 8.2	–
Fujiyoshi et al. (16)	E 7 (range 5–9) G 3 (range 2–3)	95.9 ± 32.1	4 (range 4–5)	–	82/92 (89.1%)	5.0 ± 2.5/ 1.1 ± 1.0	–	48.1 ± 17.5/	19.9 ± 13.9/ 14.6 ± 7.7	15.7 ± 9.9/ 8.6 ± 5.5	2
Sanaka et al. (17)	E 4.0 (IQR 4.0–5.0) G 4.0 (IQR 3.2–5.0) T 8.5 (IQR 8.0–9.75)	89.5 (IQR 65.2–103.7)	1.0 (IQR 1.0–1.0)	–	17/18 (94.4%)	7.0^∧^ (IQR 6.0–10.0)/0.0^∧^ (IQR 0.0–2.0)	–	–	33.4^∧^ (IQR 8.9–53.3)/ 14.2^∧^ (IQR 10.8–16.5)	15.6^∧^ (IQR 10.5–30.5)/ 3.9^∧^ (IQR 1.9–10.3)	2
Nabi Z et al. (18)	9.53 ± 1.98	62.69 ± 32.71	–	32/32 (100%)	27/32 (84.4%)	6.81 ± 1.73/ 1.18 ± 0.87	–	–	–	–	34.03 ± 13.78

*POEM, peroral endoscopic myotomy; LES, lower esophageal sphincter; IRP, integrated relaxation pressure; E, esophageal; G, gastric; T, total; IQR, interquartile range*.

∧ *Median*.

**Figure 2:**
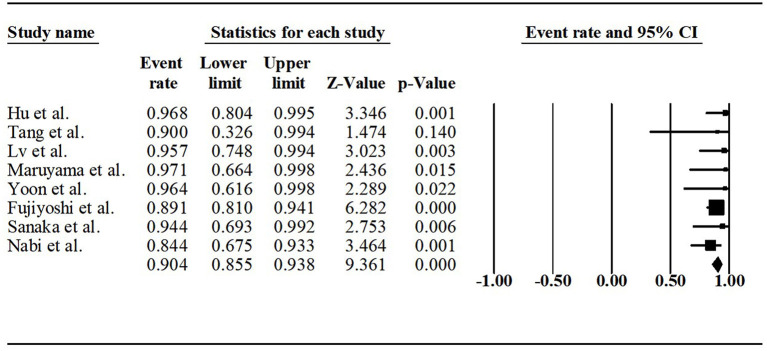
Forest plot of clinical success of POEM for sigmoid-type achalasia.

Technical success was reported in six studies. All sigmoid-type achalasia patients successfully applied POEM. Clinical success was available in all the series. Across the studies, the clinical success rate varied from 84.4 to 100%. The pooled clinical success rate was 90.4% (95% CI, 85.5%−93.8%, *I*^2^ = 0), as shown in Figure 2.

**Figure 3:**
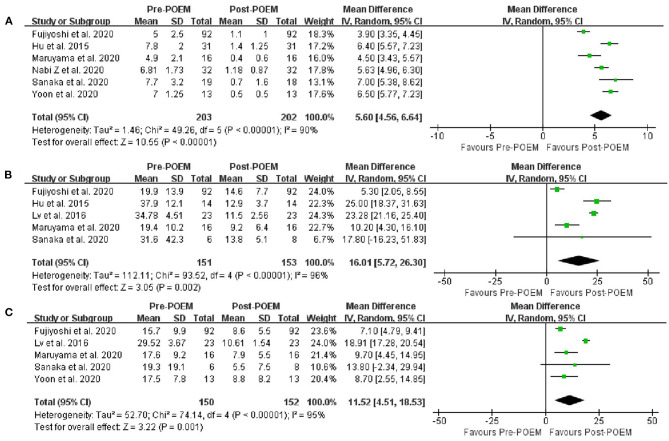
**(A)** Meta-analysis of the changes in Eckardt score after POEM in sigmoid-type achalasia. **(B)** Meta-analysis of the changes in LES pressure after POEM in sigmoid-type achalasia. **(C)** Meta-analysis of the changes in IRP after POEM in sigmoid-type achalasia.

The Eckardt score was reported in all but one series. The pre- and post-POEM Eckardt scores was significantly decreased (MD, −5.60 points; 95% CI, −4.56 to −6.64 points, *I*^2^ = 90%, *p* < 0.00001) (Figure 3A). However, there was a significant heterogeneity. The sensitivity analysis eliminated the articles of Fujiyoshi et al. (16), and the *I*^2^ decreased from 90 to 69%, MD increased from 5.60 to 5.96 points, *p* is still <0.00001. The LES pressure was reported in five articles. The pre- and post-POEM LES pressure was significantly decreased (MD, −16.01 mmHg; 95% CI, −5.72 to −26.30 mmHg, *I*^2^ = 96%, *p* = 0.02) (Figure 3B). However, there was a significant heterogeneity. However, sensitivity analysis confirmed that the result was stable. Similarly, the IRP was also reported in five articles. The pre- and post-POEM IRP was significantly decreased (MD, −11.52 mmHg; 95% CI, −4.51 to −18.53 mmHg, *I*^2^ = 95%, *p* = 0.001) (Figure 3C). There was a significant heterogeneity. The sensitivity analysis eliminated the articles of Lv et al. (13), and the *I*^2^ decreased from 95% to 0, MD decreased from −11.52 to −7.74 mmHg, and *p* decreased from 0.001 to <0.00001.

### Adverse Events and Gastroesophageal Reflux Diseases

**Figure 4:**
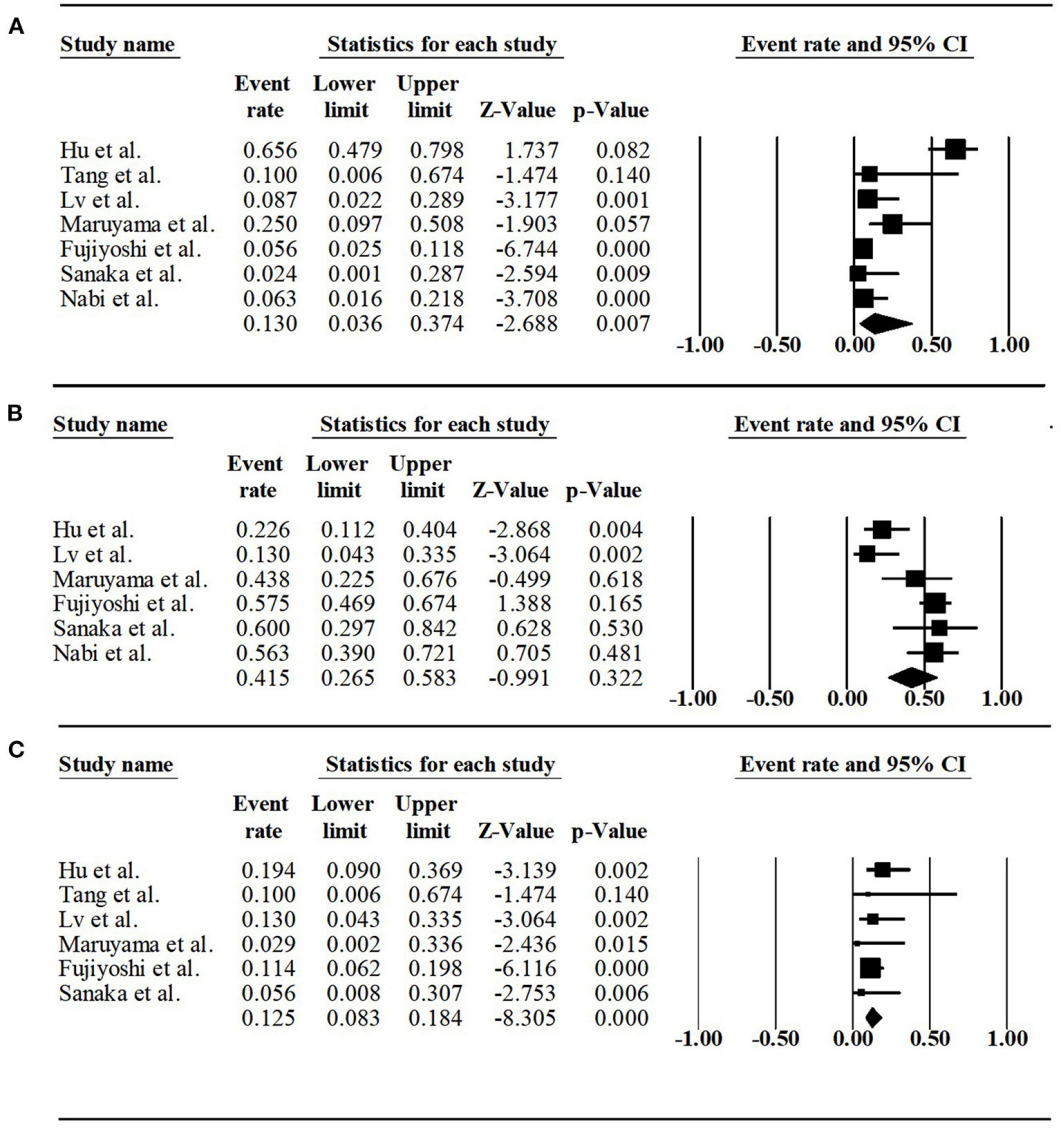
**(A)** Forest plot of adverse event rate of POEM for sigmoid-type achalasia. **(B)** Forest plot of rate of objective confirmation of reflux after POEM for sigmoid-type achalasia. **(C)** Forest plot of symptomatic reflux rate of POEM for sigmoid-type achalasia.

The prevalence of adverse events and gastroesophageal reflux diseases after POEM were summarized in Table 3. The rate of adverse events was available in all but one series. The pooled rate was 13.0% (95% CI, 3.6%−37.4%, *I*^2^ = 87.94%) (Figure 4A). There was a significant heterogeneity. The sensitivity analysis eliminated the articles of Hu et al. (6), and the *I*^2^ decreased from 87.94 to 30.31%, pooled rate decreased from 13.0 to 8.7%. The objective confirmation of reflux and symptomatic reflux were reported in all but two series. The pooled rate of objective confirmation of reflux was 41.5% (95% CI, 26.5%−58.3%, *I*^2^ = 75.54%) (Figure 4B). There was a significant heterogeneity. However, sensitivity analysis identified the stability of the pooled results. The pooled symptomatic reflux rate was 12.5% (95% CI, 8.3%−18.4%, *I*^2^ = 0).

**Table 3:** Adverse events and gastroesophageal reflux diseases after POEM.

**Study**	**Adverse events**	**Methods of diagnosis**		
		**Total**	**Objective confirmation of reflux** ** (EGD/24-h pH)**	**Symptomatic reflux**
Hu et al. (6)	Total 21/32 (mucosal injury 12; gas-related events 3; fever 6)	8/31 (25.8%)	EGD 7	6/31
Tang et al. (12)	0/4	0	–	0/4
Lv et al. (13)	Total 2/23 (gas-related events 1; perforation 1)	3/23 (13.0%)	EGD 3 (grade B)	3/23
Maruyama et al. (14)	Total 4/16 (mucosal injury 1; incomplete clipping 2; gas-related events 1)	7/16 (43.8%)	EGD 7 (grade N/A/B/C/D = 9/5/ 2/0/0)	0/16
Yoon et al. (15)	–	–	–	–
Fujiyoshi et al. (16)	Total 6/108 (perforation 3; bleeding 3)	–	EGD 50 (grade N/A/B/C/D = 37/29/ 13/7/1)	10/88
Sanaka et al. (17)	0/20	–	24-h pH 6/10	1/18
Nabi Z et al. (18)	Total 2/32 (delayed mucosal barrier failure 1; pleural effusion 1)	–	EGD 18; 24-h pH 3 (grade A/B = 7/11)	–

*POEM, peroral endoscopic myotomy; EGD, esophagogastroduodenoscopy*.

### Quality of Included Studies and Publication Bias

Supplementary Table 2 shows the quality assessment of included studies in accordance with NOS quality assessment tool. The funnel plots for the meta-analyses are illustrated in Supplementary Figure, which indicated that publication bias could not be generally considered in this meta-analysis.

## Discussion

The sigmoid-shaped achalasia is usually recognized as the advanced stage, in which the esophageal body is obviously dilated, swerved, and rotated (3). Compared with straight-shaped achalasia, sigmoid-shaped achalasia is characterized by more severe symptoms due to the morphological changes (3). POEM is a promising modality for achalasia because it is equally effective and less invasive than surgery (24). However, POEM is challenging for sigmoid-shaped achalasia. Firstly, patients with severe esophageal stasis may have submucosal inflammation and fibrosis, which hinder the establishment of submucosal tunnel. Secondly, the severe bending angle of sigmoid-shaped achalasia makes the establishment of submucosal tunnel technically challenging (15, 25). In this meta-analysis, we found that: (1) the pooled clinical success for sigmoid-type achalasia patients was 90.4%; (2) the pre- and post-POEM Eckardt scores, angle of esophageal tortuosity, diameter of esophageal, LES pressure, and IRP were significantly improved; and (3) the pooled adverse events rate was 13.0%.

So far, there is no general consensus on the most effective treatment for sigmoid-type achalasia patients. Traditionally, esophagectomy has been recommended as the primary approach because esophagectomy can remove the tortuous esophagus, while myotomy is impossible (10). However, there were many complications of esophagectomy, such as anastomotic leakage, laryngeal nerve injury, bleeding and chylothorax, pleural effusion, and cervical fistula (6). Besides, recurrent dysphagia may still be possible due to cervical esophagogastrostomic stenosis (26). It was noteworthy that the reported mortality rate for sigmoid-type achalasia, even with an experienced surgeon, was approximately 3% (26, 27). Therefore, most researchers have recommended laparoscopic Heller myotomy as a first approach for sigmoid-type achalasia in recent years (13). Many studies have also shown the effectiveness and safety of laparoscopic myotomy for sigmoid-type achalasia (9, 28). At present, POEM as a novel, minimally invasive and effective myotomy with low incidence of complications shows a special superiority.

A recent systematic review by Li et al. (29) showed that the overall clinical success rate of POEM for treatment all achalasia patients was 92.9%, the overall rate of complications was 21.2%, the rate of gastroesophageal reflux disease was 10.2% and the rate of mortality after POEM was 0, which is similar to our study. Thus, this result may suggest that POEM is equally effective in treating patients with non-sigmoid-type achalasia or sigmoid-type achalasia. However, it must be noted that POEM in the treatment of sigmoid-type achalasia is much more difficult technically than straight-shaped achalasia. Hu et al. (6) suggested that mucosal incision should be closer to the cardia and choose a relatively straight path so that the subsequent submucosal tunnel would be shorter. As the submucosal tunnel was too long, it was easy to get lost in the tunnel in such a tortuous esophagus. Lv et al. (13) demonstrated that the shorter tunnel length can reduce the difficulty of constructing the submucosal tunnel, as well as the curvature of the tunnel and might reduce the gas-related event. In such challenging procedures, another concern is associated adverse events. Mucosal perforation is more likely to occur because of the morphological changes, fibrosis, and limited space in submucosal tunnels. Another concern is related complications in such challenging procedures. Due to the morphological changes, the fibrosis, and limited space in the submucosal tunnel, mucosal perforation may happen easily in the dissection process. Hu et al. (6) reported that the rate of mucosal injury or perforations was 37.5%, which was higher than that in nonsigmoid-type achalasia (29). Therefore, POEM for sigmoid-type achalasia should be performed by an experienced operator.

Hu et al. (6) found that the esophageal lumen was still dilated in all cases during their follow-up. However, the recent research by Yoon et al. (15) reported that POEM provided morphological improvement for patients with sigmoid-type achalasia and the improvement of esophageal tortuosity may reflect a reduced esophageal burden. Overall, in our meta-analysis, the angle of esophageal tortuosity and the diameter of esophageal were significantly changed after POEM procedure.

Our meta-analysis showed that the rate of reflux was quite high, in which pooled rate of objective confirmation of reflux was 41.5% and the rate of symptomatic reflux was 12.5%. Reflux would be an inevitable problem after POEM because there was no antireflux procedure. Most patients usually have remissions with medical therapy (such as proton pump inhibitors and H2-blocking agents). Refractory reflux disease could also be further treated by endoscopic fundoplication and laparoscopic partial fundoplication, which has been reported to help alleviate the clinical reflux (30, 31).

There are some limitations to the present analysis. Firstly, there were few randomized controlled trials for meta-analysis because of the rarity of sigmoid-type achalasia. All the studies we included were retrospective or cohort studies, with two of them being presented only as published conference abstracts, which may lead to selection bias and reporting bias. Secondly, heterogeneity was noted in the pre- and post-POEM Eckardt scores, LES pressure, IRP, pooled adverse event rate, and objective confirmation of reflux rate, which may change the results. Thirdly, there were still many published papers which may have subgroup data on sigmoid-type achalasia patients. However, we cannot obtain this data by contacting the author. We can only include the eight studies in our meta-analysis, which may affect the results. Finally, despite contacting authors by email, we still cannot get individual-level data of Eckardt score from included studies and some of the articles have missing variables, which prevented us from doing more detailed and comprehensive research.

Despite these limitations, our meta-analysis provided a better understanding for the efficacy and safety of POEM in the treatment of sigmoid-type achalasia. However, a series of large-scale randomized controlled trials are still needed to prove the superiority of this technique.

## Data Availability Statement

The original contributions presented in the study are included in the article/Supplementary Material, further inquiries can be directed to the corresponding authors.

## Author Contributions

All authors listed have made a substantial, direct and intellectual contribution to the work, and approved it for publication.

## Conflict of Interest

The authors declare that the research was conducted in the absence of any commercial or financial relationships that could be construed as a potential conflict of interest.
