# Supplementary material for: Efficacy and Safety of Peroral Endoscopic Myotomy for Sigmoid-Type Achalasia: A Systematic Review and Meta-Analysis
Source: Front Med (Lausanne). 2021 Jul 8;8:677694. doi: 10.3389/fmed.2021.677694 (PMC8295649; doi:10.3389/fmed.2021.677694)
Supplement: Supplementary file 1 [file Data_Sheet_1.doc]

Table S1 Detailed search terms of each of search engines used in analysis.

| PubMed | (Esophageal Achalasia OR Achalasia Esophageal OR Esophageal Achalasia OR Cardiospasm OR Cardiospasms OR Achalasia OR Achalasias OR Achalasia, Esophageal OR Megaesophagus) AND (POEM OR Per-oral endoscopic myotomy OR Peroral endoscopic myotomy OR Per oral endoscopic myotomy) |
| --- | --- |
| Embase | (achalasia OR achalasia cardiae OR achalasia esophageal OR cardia achalasia OR esophageal achalasia OR oesophageal achalasia) AND (POEM OR Per-oral endoscopic myotomy OR Peroral endoscopic myotomy OR Per oral endoscopic myotomy) |
| Cochrane | POEM OR Per-oral endoscopic myotomy OR Peroral endoscopic myotomy OR Per oral endoscopic myotomy |

Table S2 Quality assessment of studies with NIH quality assessment tool for before-after studies with no control group.

| Study | 1 | 2 | 3 | 4 | 5 | 6 | 7 | 8 | 9 | 10 | 11 | 12 | Result |
| --- | --- | --- | --- | --- | --- | --- | --- | --- | --- | --- | --- | --- | --- |
| Hu et al. [6] | Yes | Yes | Yes | Yes | Yes | Yes | Yes | No | Yes | Yes | No | N/A | Fair |
| Tang et al. [12] | Yes | Yes | Yes | Yes | No | Yes | Yes | No | Yes | Yes | No | N/A | Fair |
| Lv et al. [13] | Yes | Yes | Yes | Yes | Yes | Yes | Yes | No | Yes | Yes | Yes | N/A | Good |
| Maruyama et al. [14] | Yes | Yes | Yes | Yes | No | Yes | Yes | No | Yes | Yes | No | N/A | Fair |
| Yoon et al. [15] | Yes | Yes | Yes | Yes | No | Yes | Yes | No | Yes | Yes | No | N/A | Fair |
| Fujiyoshi et al. [16] | Yes | Yes | Yes | Yes | Yes | Yes | Yes | No | Yes | Yes | Yes | N/A | Good |
| Sanaka et al. [17] | Yes | Yes | Yes | Yes | Yes | Yes | Yes | No | Yes | Yes | No | N/A | Fair |
| Nabi Z et al. [18] | Yes | No | Yes | Yes | Yes | Yes | Yes | No | Yes | Yes | No | N/A | Fair |

NIH, National Institutes of Health; N/A, not applicable

1. Was the study question or objective clearly stated?

2. Were eligibility/selection criteria for the study population prespecified and clearly described?

3. Were the participants in the study representative of those who would be eligible for the test/service/ intervention in the general or clinical population of interest?

4. Were all eligible participants that met the prespecified entry criteria enrolled?

5. Was the sample size sufficiently large to provide confidence in the findings?

6. Was the test/service/intervention clearly described and delivered consistently across the study population?

7. Were the outcome measures prespecified, clearly defined, valid, reliable, and assessed consistently across all study participants?

8. Were the people assessing the outcomes blinded to the participants’ exposures/interventions?

9. Was the loss to follow-up after baseline 20% or less? Were those lost to follow-up accounted for in the analysis?

10. Did the statistical methods examine changes in outcome measures from before to after the intervention? Were statistical tests done that provided P values for the pre-to-post changes?

11. Were outcome measures of interest taken multiple times before the intervention and multiple times after the intervention?

12. If the intervention was conducted at a group level, did the statistical analysis take into account the use of individual-level data to determine effects at the group level?
