# Supplementary figures and images for: Efficacy and Safety of Peroral Endoscopic Myotomy for Sigmoid-Type Achalasia: A Systematic Review and Meta-Analysis
Source: Front Med (Lausanne). 2021 Jul 8;8:677694. doi: 10.3389/fmed.2021.677694 (PMC8295649; doi:10.3389/fmed.2021.677694)

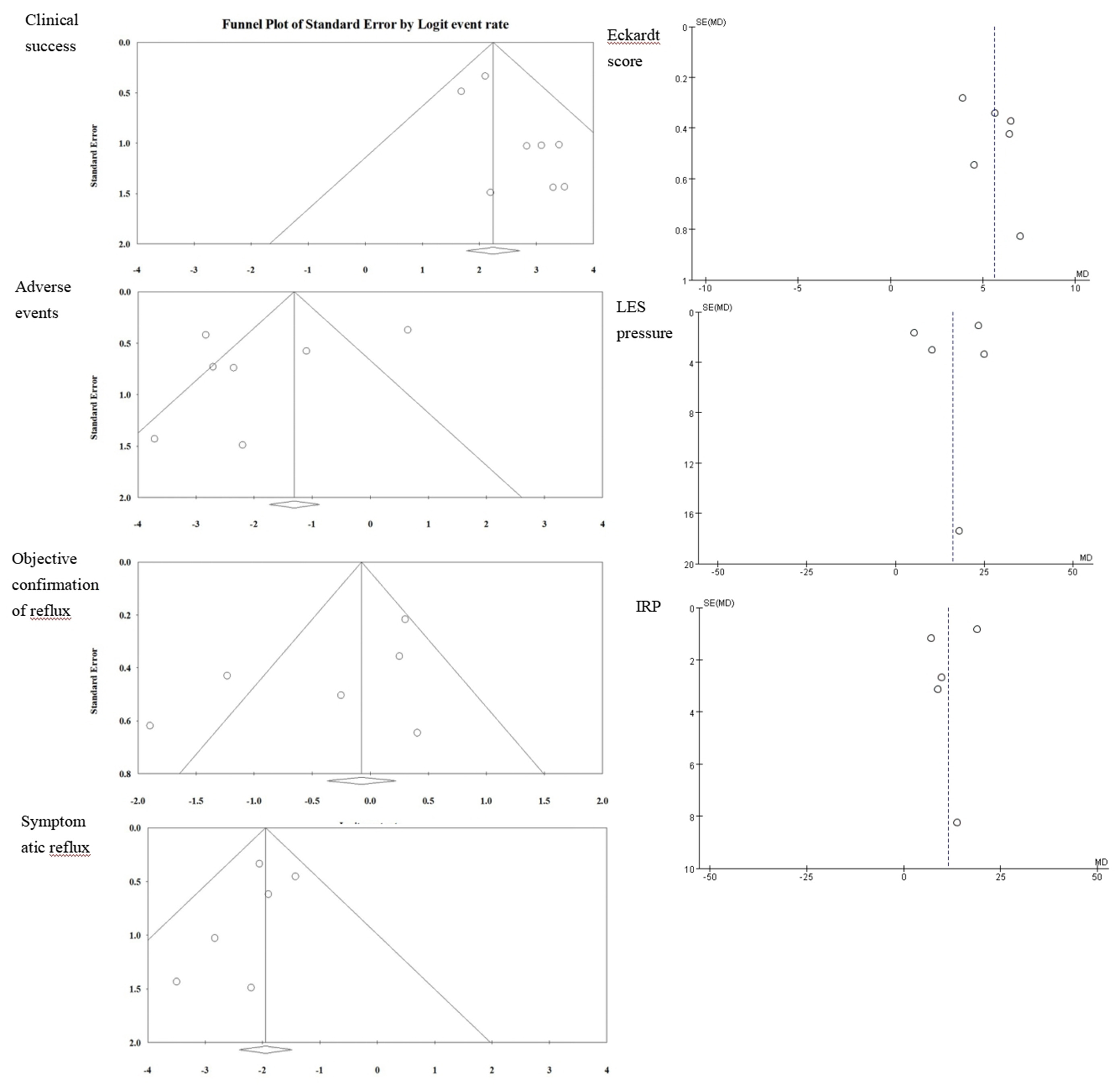

Supplement: Supplementary file 2 [file Image_1.JPEG]
